# Prospective blinded clinical evaluation of a fully automated workflow for prostate radiotherapy

**DOI:** 10.1016/j.phro.2026.100999

**Published:** 2026-05-16

**Authors:** David Neugebauer, Jan-Hendrik Bolten, Hoi H. Lau, Stephan Mende, Tilmann Rackwitz, Thomas Held, Christoph Grott, Johanna Rademacher, Fabian Weykamp, Eva Meixner, Philipp Hoegen-Sassmannshausen, Jürgen Debus, Sebastian Klüter, Jakob Liermann

**Affiliations:** a Klinik für Radioonkologie und Strahlentherapie, Universitätsklinikum Heidelberg, Germany; b Heidelberg Institute of Radiation Oncology (HIRO), Universitätsklinikum Heidelberg, Germany; c Nationales Zentrum für Tumorerkrankungen (NCT), Heidelberg, Germany; d Klinische Kooperationseinheit Strahlentherapie, Deutsches Krebsforschungszentrum (DKFZ), Heidelberg, Germany

**Keywords:** Automation, Treatment planning, Auto-contouring, Auto-planning, Artificial intelligence, Prostate Cancer, Image-Guided radiotherapy, VMAT

## Abstract

**Background and purpose:**

Fully automated workflows integrating deep learning–based segmentation and treatment planning have shown promising results in research settings but require prospective evaluation under routine clinical conditions. This study assessed the clinical usability of a fully automated workflow for prostate radiotherapy compared with the conventional clinical workflow.

**Materials and Methods:**

Twenty-two consecutively treated patients undergoing prostate radiotherapy were included. For each patient, treatment planning was first performed according to the conventional workflow, followed by execution of a fully automated workflow combining deep learning–based segmentation and planning models within a commercial treatment planning system. Plans and structure sets were evaluated in a blinded, standardized manner by board-certified radiation oncologists and rated as “good”, “acceptable”, or “unacceptable”. The preferred plan was selected for treatment. Processing times were recorded.

**Results:**

Clinically acceptable results were achieved in 86% of fully automated cases compared with 95% in the conventional workflow. Conventional plans were rated more frequently as “good” (17/22 vs 12/22), mainly due to differences in target delineation. Fully automated clinical target volumes were consistently smaller (median 54.2 cm^3^ vs 68.3 cm^3^; p < 0.001). The fully automated workflow required a median of 9 min (range 8–10) compared with 87 min (range 70–130) for the conventional workflow.

**Conclusions:**

A fully automated workflow for prostate radiotherapy substantially reduces workload and processing time while achieving a high rate of clinical acceptability. Implementation as a draft-first approach with mandatory physician review appears feasible, although refinement of automated target delineation is warranted.

## Highlights

• Fully automated and standard workflows were prospectively compared.
• Full automation achieved clinically acceptable results in 86% of 22 patients.
• Total preparation time decreased from 87 min to about 9 min.
• Standard workflow preferred mainly due to smaller automated targets.
• Full automation reduces workload with mandatory physician review.

## Graphical abstract

**Figure:**
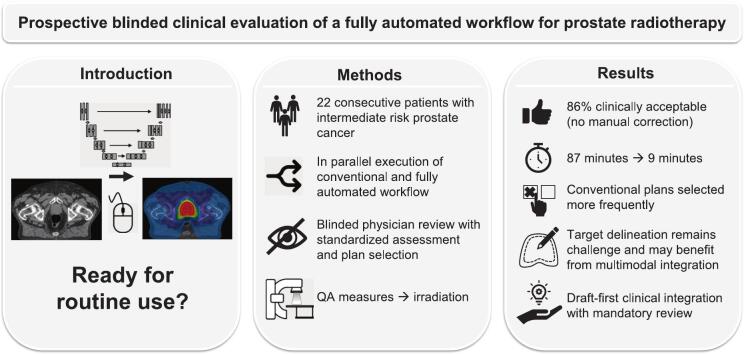

## 1 Introduction

Machine learning techniques (ML) in radiotherapy for contouring and plan creation are generally based on a subjective, human-derived ground truth [1]. As a result, their performance is closely tied to the quality of this input and may therefore mimic rather than surpass human-driven results [2]. With regard to automated treatment planning, the absence of a well-defined optimal solution presents an additional challenge. Consequently, the main advantage of ML lies in increased efficiency, which is urgently needed [3] due to the growing demand for individualized and adaptive radiotherapy, as conventional segmentation and inverse treatment planning remain among the most time-consuming steps in radiotherapy workflows [4]. In particular, the expansion of online adaptive radiotherapy is difficult to envisage without ML because of strict time constraints. Another advantage is the mitigation of inter-observer variability in segmentation [5], [6] and planning [7]. Driven by these benefits, automation through ML in radiotherapy has received considerable research attention and has made substantial progress, producing predominantly clinically acceptable results for both automatic segmentation [8], [9], [10] and planning [11], [12], [13] in numerous studies.

As a consequence, an increasing number of ML-based segmentation and planning tools have transitioned from research into clinical routine [14], [15]. This development raises the frequently discussed question [16], [17] of how close the field is to achieving a fully automated workflow (FAW) when these two components are combined. A recent challenge by Gooding et al. [18] demonstrated that FAWs can produce clinically acceptable results but also highlighted a lack of trust, particularly due to missing comparisons with conventional plans. To address the need for prospective clinical evidence, we conducted a blinded clinical study building on the work of Bolten et al. [19], who previously assessed the feasibility of our in-house-developed FAW. The present study was deliberately designed to mirror real clinical workflows as closely as possible, accepting the inherent variability and practical constraints associated with such a realistic setting.

## 2 Materials and methods

### 2.1 Study design and patient cohort

This prospective clinical study included 22 consecutive patients with localized prostate cancer treated with definitive prostate radiotherapy at our institution in 2024. Patients were excluded only if predefined exclusion criteria were met. Prescribed doses were 60 Gy in 20 fractions or 76.5 Gy in 34 fractions. Inclusion of seminal vesicles in the target volume was based on the patient-specific risk profile, and delineation of the seminal vesicles was performed in accordance with the ESTRO ACROP consensus guidelines [20].

Exclusion criteria comprised total hip replacement implants, prior transurethral resection of the prostate (TURP), or previous high-intensity focused ultrasound therapy (HIFU), as these interventions may substantially alter pelvic anatomy.

The study was approved by the Ethics Committee of Heidelberg University (S-193/2024).

### 2.2 Clinical and fully automated workflows

**Conventional workflow (CW)**.

After import of the planning CT, organs of interest (OOIs) were contoured by randomly assigned radiation therapy technologists (RTTs) and reviewed by a board-certified radiation oncologist, who subsequently delineated the clinical target volume (CTV). All available imaging could be used for contouring. Magnetic resonance imaging was available for 10 of 22 patients and PSMA-PET/CT for 5 of 22 patients.

OOI contours in routine clinical practice were initially generated using deep learning–based segmentation (DLS) and manually adapted when required. Following target delineation, randomly assigned RTTs generated volumetric-modulated arc therapy (VMAT) plans using iterative optimization parameter selection. This process corresponded to the standard in-house clinical workflow. The resulting structure set and treatment plan are hereafter referred to as the conventional workflow (CW).

**Fully automated workflow (FAW)**.

After completion of the CW, a fully automated workflow was executed to avoid influencing RTT planning. A dedicated in-house script with a graphical user interface was initiated via the treatment planning system (TPS) scripting interface after selection of the risk profile and dose concept. No manual interaction was required after initialization, and the generated plan was not further optimized, scaled, or adapted.

Both CW and FAW plans were generated as VMAT treatments with two full arcs, 6 MV photon energy, and 4° gantry spacing. FAW contouring relied exclusively on the planning CT.

### 2.3 Deep learning segmentation and machine-learning planning models

All structure delineation and treatment planning were performed in RayStation version 11B (RaySearch Laboratories AB, Stockholm, Sweden).

Automatic contouring used the DLS model *Male Pelvic CT 11B* (v1.0.0.7, RaySearch) integrated within the TPS. Customization was limited to structure naming and type assignment; generated geometries could not be modified algorithmically but could be manually edited within the TPS after creation.

Automated plan generation employed the *RSL-Prostate-6000* model (RaySearch), which combines machine-learning-based spatial dose prediction with subsequent dose mimicking [21]. The model contains a fixed training dataset but was clinically configured for each prescription concept regarding post-processing of predicted dose and dose-mimicking parameters.

### 2.4 Plan evaluation and endpoints

Plan and structure identifiers were randomized prior to evaluation to ensure blinded assessment while preserving correspondence between each plan and its structure set. OOIs routinely delineated clinically but not generated by the DLS model (penile bulb, small bowel, sigmoid) were labeled without workflow attribution.

**Fig. 1:**
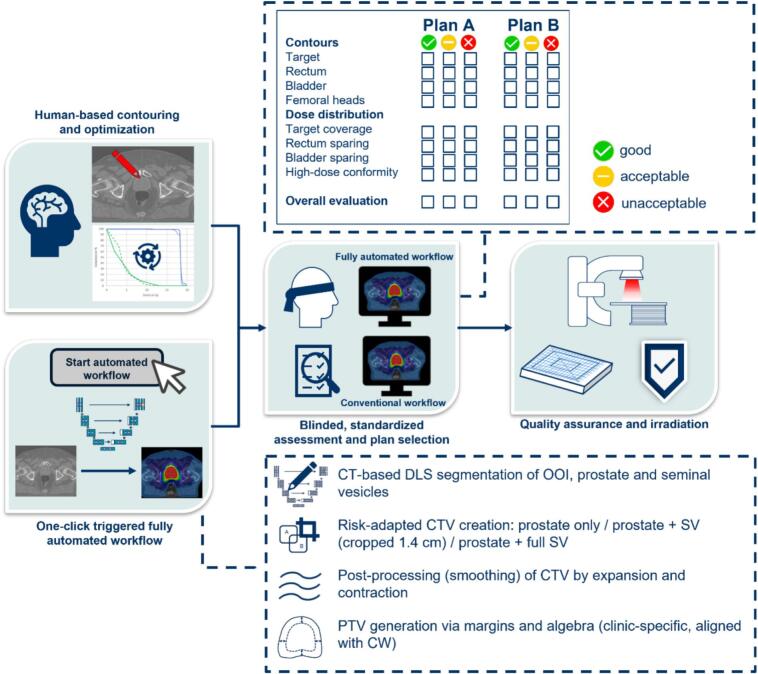
Overview of the study design and clinical workflows. The fully automated workflow (FAW) integrates deep learning–based segmentation and machine-learning–based treatment planning within the treatment planning system and is executed in parallel with the conventional workflow (CW). Resulting structure sets and plans undergo blinded physician evaluation, standardized assessment, and clinical plan selection prior to quality assurance and treatment delivery.

Evaluations were performed by multiple board-certified radiation oncologists who had not delineated the CW targets and were randomly assigned to cases. Assessment followed a predefined standardized form (Fig. 1). Because the structure sets differed, a purely numerical DVH comparison would have introduced bias. We therefore applied a predefined structured qualitative evaluation reflecting routine clinical plan assessment, including evaluation of gradients and isodose shapes in addition to compliance with standard dose guidance.

Ratings were defined as follows:

• **Good**: high agreement with the physician’s clinical judgment.

• **Acceptable**: clinically approvable for treatment despite minor preferred adjustments.

• **Unacceptable**: potential to negatively impact treatment outcome.

The primary endpoint was the proportion of FAW plans rated clinically acceptable. Secondary endpoints included physician preference between FAW and CW plans, as well as geometric similarity between CW- and FAW-derived CTVs and PTVs. Similarity metrics comprised Dice similarity coefficient, mean distance to agreement, Hausdorff distance, and sensitivity. Paired comparisons of CTV volumes were performed using the Wilcoxon signed-rank test.

Statistical analyses were performed using Python (version 3.9.13).

## 3 Results

In 91% of patients, FAW target volumes were rated clinically acceptable or better, with 45% rated as “good.” In the CW, 64% of targets were rated as “good” and 36% as “acceptable.” For OOIs, no CW structure was rated as “unacceptable.” In contrast, FAW OOI ratings included a small number of “unacceptable” cases, and the proportion of structures not rated as “good” was higher than in the CW.

**Fig. 2:**
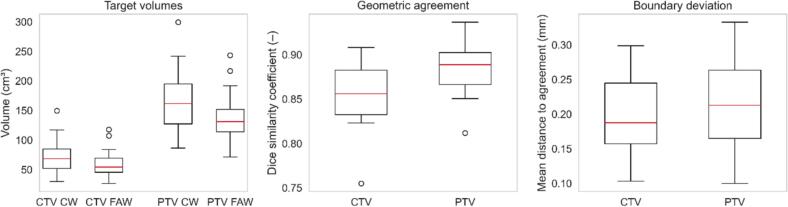
Target volumes and geometric agreement between conventional and fully automated workflows. Boxplots display median, interquartile range, whiskers (1.5 × IQR), and outliers. Volume (cm^3^), Dice similarity coefficient (–), and mean distance to agreement (mm) are shown for CTV and PTV.

Automatically generated target volumes were consistently smaller, with a median FAW CTV volume of 54.2 cm^3^ (range 26.4–117.6 cm^3^) compared with 68.3 cm^3^ (range 29.7–149.4 cm^3^) in the CW (Wilcoxon signed-rank test, p < 0.001; Fig. 2), and were occasionally considered too short in the caudal direction. In some patients, the delineation of the seminal vesicle base was judged too short, and minor inaccuracies in the demarcation between bladder and prostate were observed.

Median Dice similarity coefficient for CTVs was 0.86 (range 0.76–0.91), with a mean distance to agreement of 0.19 mm (range 0.10–0.30 mm) (Table 1).

**Table 1:** Geometric similarity metrics between CW- and FAW-derived CTVs and PTVs.

		DSC	Mean DTA (mm)	Hausdorff (mm)	Sensitivity
PTV	mean ± SD	0.89 ± 0.03	0.21 ± 0.06	1.08 ± 0.34	0.81 ± 0.05
	median (range)	0.89 (0.81–0.94)	0.21 (0.10–0.33)	1.10 (0.52–1.63)	0.82 (0.69–0.90)
CTV	mean ± SD	0.86 ± 0.04	0.19 ± 0.06	1.07 ± 0.34	0.78 ± 0.06
	median (range)	0.86 (0.76–0.91)	0.19 (0.10–0.30)	1.04 (0.56–1.64)	0.77 (0.62–0.87)

All FAW plans achieved “good” target coverage, compared with 86% in the CW (Table 2). High-dose conformity was rated “good” in 95% of FAW plans and 82% of CW plans. Rectum sparing was rated “good” more frequently in the CW (91%) than in the FAW (73%). Overall, FAW dose distributions were evaluated positively, with no unacceptable dose-distribution criteria and the majority rated as “good.” Additional dose-volume metrics are provided in the Supplementary Material.

**Table 2:** Standardized clinical evaluation of structure sets, dose distributions, and overall plan quality for CW and FAW.

	Conventional workflow (CW)			Fully automated workflow (FAW)		
Structure set						
	good	acceptable	unacceptable	good	acceptable	unacceptable
Target	14	8	0	10	10	2
Rectum	21	1	0	15	5	2
Bladder	22	0	0	17	4	1
Femoral Heads	18	4	0	17	5	0
Dose distribution						
Target coverage	19	3	0	22	0	0
Rectum sparing	20	2	0	16	6	0
Bladder sparing	17	5	0	19	3	0
High dose conformity	18	4	0	21	1	0
Complete plan						
Overall assessment	17	4	1	12	7	3
	Conventional Workflow		Fully automated workflow		Equivalent	Both rejected
Final plan selection	11		4		6	1

Overall clinically acceptable plans were achieved in 86% of FAW cases and 95% of CW cases. Plans rated as “good” accounted for 55% in the FAW and 77% in the CW, while “unacceptable” ratings occurred in 14% and 5%, respectively. Final clinical plan selection favored the CW in a larger number of cases (Table 2).

In eight patients, proximity of the sigmoid or small bowel to the target required explicit contouring for dose assessment. In three patients, FAW target volumes overlapped with an OOI, leading to conflicts between target coverage and clinic-defined dose limits. In these cases, FAW plans were rated “unacceptable” because the ML planning model was not adapted to such anatomical situations. For one of these patients, the CW plan was also rejected to improve OOI sparing.

For the CW, mean times were 20 min for OOI contouring, 37 min for physician review and target delineation, and 31 min for treatment planning, resulting in a total mean workflow time of 87 min (range 70–130 min).

In contrast, the FAW required a mean total time of 9 min (range 8–10 min), excluding additional manual contouring of structures not available in the auto-segmentation model.

None of the selected plans, whether from CW or FAW, failed patient-specific quality assurance following plan selection.

## 4 Discussion

In this prospective clinical study, we implemented a FAW integrating deep learning–based segmentation and planning within a commercial TPS for prostate radiotherapy. Automated plans were generated in parallel with the CW and evaluated in a blinded, standardized manner under routine clinical conditions. The FAW achieved a high rate of clinical acceptability, with most plans meeting clinical requirements without manual intervention, demonstrating feasibility for routine clinical integration. Physicians nevertheless selected CW plans more often, mainly due to smaller target volumes and slightly less pronounced rectum sparing in FAW plans.

Previous studies have predominantly focused on automated segmentation or automated treatment planning as isolated components, often emphasizing geometric accuracy or dose distribution characteristics under controlled experimental conditions. In contrast, the present work evaluated an end-to-end FAW embedded in routine clinical practice, encompassing segmentation, planning, and clinical decision-making.

In principle, the agreement between FAW- and CW-derived CTVs, as reflected by Dice similarity coefficients, lay within the range reported in existing literature [22], [23]. At the same time, a substantial proportion of conventionally contoured target volumes were not rated as “good” but rather as “acceptable,” further underscoring the well-known inter-observer variability in prostate target delineation [5], [6]. Against this background, the observed differences between manually and automatically contoured target volumes should be interpreted with caution. While a systematic tendency toward smaller automatically contoured targets was observed, this finding does not necessarily indicate inferior contour quality. Rather, it likely reflects differences in contouring approach and available information, as well as potential variations in institutional practice underlying model development. These observations highlight the need for further investigation of systematic volume differences during clinical implementation and emphasize the importance of careful structure evaluation for each individual patient.

FAW dose distributions were comparable in quality but showed slightly different trade-offs between target conformity and OOI sparing despite alignment with conventional in-house dose distributions, underscoring the role of institutional conventions and planner bias [24], [25]. In this context, automated planning offers an opportunity to reassess established planning strategies and promote greater consistency.

In routine practice, physicians frequently evaluate a single plan and may not have performed the target delineation themselves. Under these conditions, the task is to determine whether the proposed structure set and dose distribution are clinically acceptable rather than whether they precisely match individual preferences. The high proportion of FAW plans deemed acceptable supports the suitability of the FAW for routine clinical use, with plan rejection primarily attributable to identifiable segmentation limitations rather than deficiencies in planning quality. Moreover, the clinical relevance of highly detailed contour refinement on the planning CT should be interpreted in the context of daily anatomical variation during treatment, which may limit the practical impact of small geometric differences between contouring approaches.

A major strength of this study is its prospective evaluation of a FAW under routine clinical conditions, involving multiple RTTs and physicians. Integration into daily practice with blinded plan evaluation captured inter-observer variability and real-world clinical decision-making often underrepresented in controlled benchmarking studies. The parallel generation of FAW and CW plans enabled a realistic assessment of clinical feasibility, with CW plans serving as an objective clinical reference and helping to calibrate confidence in automated solutions.

Several limitations should be acknowledged. First, the comparison of target volume delineation between the FAW and the CW is not entirely equivalent. While the FAW relies exclusively on the planning CT, target delineation in the CW incorporated additional imaging modalities, such as magnetic resonance imaging or prior diagnostic studies, when available. Although this reflected current clinical practice, it may have disadvantaged the FAW in direct geometric comparisons. Second, the automated segmentation tool used in this study did not include certain OOIs, such as the sigmoid and bowel loops, which contributed to FAW plan rejection in selected cases. Finally, the single-institution design and use of a specific treatment planning system with an in-house developed FAW script may limit generalizability and warrant multi-institutional validation.

At the current stage, the FAW is only applicable to anatomically well-defined targets that can be reliably delineated on planning CT alone. The integration of multimodal imaging and the incorporation of text-based clinical information, for example through large language models [26], may substantially expand the applicability of FAWs and further improve segmentation accuracy [27].

In contrast to auto-segmentation or auto-planning alone, a FAW represents a promising innovation with the potential to reshape the clinical workflow rather than merely accelerate individual steps. A near-perfect success rate is not required for clinical benefit, as occasional segmentation failures mainly result in limited computational overhead. Successful cases proceed through an accelerated and largely autonomous workflow. This balance between efficiency gains and acceptable failure tolerance supports clinical integration of the FAW as a draft-first workflow requiring mandatory physician review and explicit safety checks, while further improvements in automated segmentation remain necessary.

Safe implementation therefore requires safeguards ensuring systematic physician review of FAW-generated target volumes before treatment approval, as undersized CTVs may lead to target underdosage. These may include standardized nomenclature, explicit plan status labeling, mandatory review checkpoints, and additional verification steps according to local clinical practice. These requirements reflect responsible workflow integration rather than technical limitations of the FAW itself.

**Fig. 3:**
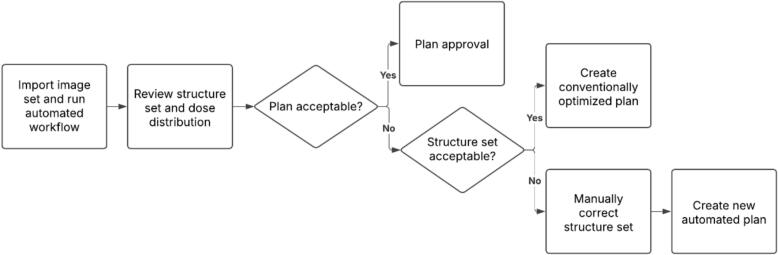
Proposed clinical decision workflow for integration of the fully automated workflow (FAW). After automated plan generation, structure set and dose distribution undergo physician review. Clinically acceptable plans proceed to approval, whereas unacceptable dose distributions trigger conventional replanning and unacceptable structures require manual correction followed by renewed automated planning.

Based on these considerations, we propose a clinical workflow in which physicians first assess a FAW-generated plan proposal upon image review. The structure set is reviewed first, followed by dose evaluation if contours are acceptable. If the plan is rejected due to defective structures, the contours are corrected and a new deep learning–based plan is generated. If the structure set is acceptable but the dose distribution is not, a new plan is created using conventional optimization (Fig. 3).

In conclusion, the present study demonstrates that a FAW for prostate radiotherapy can be integrated into routine clinical practice with a high rate of clinically acceptable results. The workflow showed substantial potential to streamline clinical planning processes while maintaining safety. Further improvements in automated segmentation and validation across institutions will be important for broader clinical implementation.

## 5 Declaration of generative AI and AI-assisted technologies in the manuscript preparation process

During the preparation of this work the authors used ChatGPT-5.2 for language editing and clarity. After using this tool, the authors reviewed and edited the content as needed and take full responsibility for the content of the publication.

## CRediT authorship contribution statement

**David Neugebauer:** Writing – original draft, Software, Methodology, Formal analysis, Conceptualization. **Jan-Hendrik Bolten:** Writing – review & editing, Data curation. **Hoi H. Lau:** Investigation, Writing – review & editing. **Stephan Mende:** Writing – review & editing, Investigation, Data curation. **Tilmann Rackwitz:** Writing – review & editing, Investigation, Data curation. **Thomas Held:** Writing – review & editing, Investigation, Data curation. **Christoph Grott:** Writing – review & editing, Data curation. **Johanna Rademacher:** Writing – review & editing, Investigation, Data curation. **Fabian Weykamp:** Writing – review & editing, Data curation. **Eva Meixner:** Writing – review & editing, Data curation. **Philipp Hoegen-Sassmannshausen:** Writing – review & editing, Data curation. **Jürgen Debus:** Writing – review & editing, Supervision, Resources, Project administration. **Sebastian Klüter:** Writing – review & editing, Supervision, Project administration. **Jakob Liermann:** Conceptualization, Supervision, Methodology, Writing – original draft, Project administration.

## Declaration of competing interest

The authors declare the following financial interests/personal relationships which may be considered as potential competing interests: DN received travel reimbursement from RaySearch Laboratories AB outside the submitted work. FW received speaker fees from AstraZeneca, Varian Medical Systems, Siemens Healthineers, Chulabhorn Royal Academy, and Merck Sharp & Dohme; travel support for attending meetings from AstraZeneca, Varian Medical Systems, Novocure GmbH, German Center for Lung Research (DZL), Fraunhofer MEVIS, Chulabhorn Royal Academy, and Micropos Medical; as well as compensation for advisory board activities from Novocure GmbH and Merck Sharp & Dohme, outside the submitted work. JD received grants or contracts from Accuray Incorporated, RaySearch Laboratories AB, Vision RT Ltd., Merck Serono GmbH, Siemens Healthcare GmbH, and PTW-Freiburg Dr. Pychlau GmbH, as well as equipment from IntraOp Medical, outside the submitted work. SK received speaker fees from ViewRay Inc. outside the submitted work. JL received travel reimbursement from RaySearch Laboratories AB, Varian Medical Systems, and Micropos Medical, as well as speaker fees from Accuray Incorporated and AstraZeneca GmbH, outside the submitted work. JL is a member of the Clinical Advisory Board of RaySearch Laboratories AB.
