# Supplementary material for: Prospective blinded clinical evaluation of a fully automated workflow for prostate radiotherapy
Source: Phys Imaging Radiat Oncol. 2026 May 16;39:100999. doi: 10.1016/j.phro.2026.100999 (PMC13224377; doi:10.1016/j.phro.2026.100999)
Supplement: Supplementary Data 1 [file mmc1.pdf]

Supplementary material

Abbreviations

CW – Conventional Workflow

FAW – Fully automated workflow

Table S1 - Clinical goals used for different fractionation schemes.

	76.5 Gy in 34 fractions	60.0 Gy in 20 fractions
PTV	D95 > 72.68Gy	D95 > 57.0Gy
Body	D0.03ccm < 81.85Gy	D0.03ccm < 64.2Gy
Rectum	D0.03ccm < 78.0Gy	D0.03ccm < 61.0Gy
	V75Gy < 15.0%	V60Gy < 15.0%
	V70Gy < 20.0%	V52Gy < 20.0%
	V65Gy < 35.0%	V47Gy < 25.0%
	V50Gy < 50.0%	V40Gy < 35.0%
		V35Gy < 50.0%
Bladder	Dmean < 35.0Gy	Dmean < 32.0Gy
	D0.03ccm < 78.0Gy	D0.03ccm < 61.0Gy
	V77Gy < 15.0%	V60Gy < 15.0%
	V75Gy < 25.0%	V57Gy < 25.0%
	V70Gy < 35.0%	V52Gy < 35.0%
	V65Gy < 50.0%	V47Gy < 50.0%
Sigmoid	D0.03ccm < 58.0Gy	D0.03ccm < 53.0Gy
Small bowel	D0.03ccm < 52.0Gy	D0.03ccm < 48.0Gy
Femoral heads	Dmean < 25.0Gy	Dmean < 20.0Gy

Table S2 - Comparison of the total number of monitor units.

	FAW	CW
76.5 Gy in 34 fractions	667.4 ± 36.1 MU	727.6 ± 79.3 MU
60 Gy in 20 fractions	925.5 ± 35.3 MU	971.4 ± 88.1 MU

Table S3a - Dosimetric comparison of CW and FAW for patients with a total dose of 76.5Gy.

Structure	Parameter	FAW	CW
PTV	V95%	0.987 ± 0.003	0.973 ± 0.004
Rectum	V75Gy	0.058 ± 0.021	0.057 ± 0.027
Rectum	V70Gy	0.117 ± 0.04	0.104 ± 0.042
Rectum	V65Gy	0.154 ± 0.048	0.135 ± 0.050
Rectum	V50Gy	0.240 ± 0.062	0.215 ± 0.064
Rectum	mean	28.93 ± 4.63 Gy	27.39 ± 5.31 Gy
Bladder	V75Gy	0.058 ± 0.021	0.040 ± 0.021
Bladder	V70Gy	0.057 ± 0.039	0.067 ± 0.039
Bladder	V65Gy	0.072 ± 0.049	0.083 ± 0.048
Bladder	mean	16.73 ± 8.82 Gy	19.15 ± 7.97 Gy

Table S3b - Dosimetric comparison of CW and FAW for patients with a total dose of 60.0Gy.

Structure	Parameter	FAW	CW
PTV	V95%	0.991 ± 0.002	0.976 ± 0.002
Rectum	V60Gy	0.010 ± 0.007	0.015 ± 0.014
Rectum	V52Gy	0.142 ± 0.043	0.129 ± 0.041
Rectum	V47Gy	0.180 ± 0.052	0.164 ± 0.050
Rectum	V40Gy	0.227 ± 0.062	0.208 ± 0.059
Rectum	V35Gy	0.259 ± 0.068	0.240 ± 0.065
Rectum	mean	21.88 ± 3.48 Gy	21.47 ± 3.63 Gy
Bladder	V57Gy	0.064 ± 0.038	0.069 ± 0.043
Bladder	V52Gy	0.091 ± 0.054	0.098 ± 0.063
Bladder	V47Gy	0.111 ± 0.067	0.122 ± 0.078
Bladder	mean	15.07 ± 6.57 Gy	16.83 ± 7.11 Gy
